# A Hand-Book of Ophthalmic Science and Practice

**Published:** 1885-03

**Authors:** 


**A Hand-Book of Ophthalmic Science and Practice.** By Henry C. Juler., F. R. C. S., Ophthalmic Surgeon to St. Mary's Hospital and to the Royal Westminster Ophthalmic Hospital. With 125 illustrations. Philadelphia: H. C. Lea's Son & Co. 1884.

We have here a most admirable book. We can heartily commend it as one which will fitly meet the requirements of all those who desire to possess a reliable guide in this branch of the science of medicine. The reader will find not only clear and concise descriptions of all the important affections of the eye, but also colored plates really beautiful in execution, which, to those who have not had the advantage of study in large ophthalmic hospitals will be of the greatest service in the study of diseases of the eye. Ludlow's and Jaeger's test types are introduced at the end of the volume. The general practitioner, particularly, will find in this book the kind of knowledge he most needs in his practice. In every respect the work reflects credit on its author. We have not seen the English edition, but as regards type work and illustrations, we believe that this book could hardly be surpassed in beauty of execution.

